# Dealing with soft variables and data scarcity: lessons learnt from quantification in a participatory system dynamics modelling process

**DOI:** 10.1002/sdr.1770

**Published:** 2024-04-14

**Authors:** Irene Pluchinotta, Ke Zhou, Nici Zimmermann

**Affiliations:** Institute for Environmental Design and Engineering, The Bartlett Faculty of The Built Environment, University College London, London, UK

## Abstract

System dynamics (SD) models are commonly used for structuring complex problems to support decision-making. They are used to investigate areas in which limited knowledge is available, describing nonlinear relationships and including intangible elements. Although this explorative nature is one of the key advantages, it also represents a challenge for quantifying the intangible, i.e. more qualitative aspects of an SD model, especially when it is not possible to apply conventional analytical methods due to data scarcity. Procedures to obtain and analyse information using participatory approaches are limited. First, this article outlines existing quantification methods and related open questions when dealing with soft variables and data scarcity. Secondly, it summarises the quantification process developed during a participatory SD process, describing how we dealt with data scarcity and soft variables. Lastly, we suggest a quantification framework in relation to data availability and level of stakeholder engagement.

## Introduction

System dynamics (SD) simulation models are commonly used for exploring, structuring, and managing complex problems in order to design more effective policies and support decision-making. They are often used to investigate areas in which limited knowledge is available, describing nonlinear relationships and including variables representing intangible elements of the system (Sterman, 2000). Indeed, SD practitioners and researchers use formal simulation models to overcome cognitive limitations, to grasp the dynamic complexity of a problem situation, and to make reliable inferences about system behaviour (Oliva, 2003). While this explorative nature is a key advantage of SD models, it also represents a major challenge for modellers working on the quantification and parametrisation of the qualitative aspects of a model, namely soft and intangible variables and data-scarce contexts, especially when it is not possible to apply conventional analytical methods (e.g. the ones described in Rahmandad *et al*., 2015). This is notably prevalent in participatory modelling processes. Although there is general agreement about the importance of qualitative data during the development of an SD model, there is a limited availability of procedures to obtain and analyse qualitative information (Luna-Reyes and Andersen, 2003), especially in a participatory setting.

SD modellers depend on expert and local knowledge, often collected via processes managed and operated by interdisciplinary teams (Ford and Sterman, 1998) to structure and parameterise a *useful* model (Oliva, 2003). However, as underlined by Ford and Sterman (1998), while many methods to elicit information from experts have been developed, most assist in the early phases of conceptual modelling (i.e. problem articulation, boundary selection, identification of variables, and qualitative causal mapping); the scientific literature offers fewer methods to elicit the information required to estimate the parameters, initial conditions, and quantify relationships, all of which must be specified in formal modelling. In some cases, the uncertainty associated with the quantification of soft variables has caused experts to believe that the results from ensuing simulations could be misleading, or at least, very fragile (Coyle, 2000).

In participatory modelling processes, it is often the modelling team who conducts the quantification and parameter estimation after the Group Model Building (GMB) sessions without much input from participants (Hosseinichimeh *et al*., 2017). Furthermore, Hosseinichimeh *et al*. (2016) highlight that there is limited methodological guidance for estimating values and quantifying relationships in SD models using datasets common to social sciences that include few data points over time, thus emphasising the need for more rigorous estimation methods in the SD literature (with the exception of Hosseinichimeh *et al*., 2017). Indeed, excluding the more qualitative aspects from an SD simulation model due to the lack of knowledge likely implies neglecting major influences in the system (Forrester, 1971). In practice, SD modellers face a recurrent question on how to deal with the quantification of the qualitative aspects of an SD model especially through participatory methods, namely, how to deal with soft variables and how to carry out a quantification or parameter estimation process when there are no data available (Dianati *et al*., 2019). This modelling issue is stressed when facing complex socioenvironmental problems.

Within this context, the objective of this article is to suggest a quantification framework providing guidance on how different types of data can be elicited in relation to data availability and to the level of stakeholder engagement (the framework is presented in Table 3 and Figure 5). As a contribution to the SD community, the framework shows the breadth of data modellers can use to quantify a model and strategies we can employ when data are not available. Building up from existing methods and adapting them under an operational perspective, the framework is based on the quantification process developed during a participatory modelling activity on a complex environmental issue (namely the use of natural space in Thamesmead, London, United Kingdom). When describing our quantification process, we focus on how we managed the scarcity of data and how we dealt with the presence of a high number of soft variables.

To develop the framework, we also explored existing quantification methods and open questions when dealing with soft and intangible variables and data scarcity during participatory processes. We use “data scarcity” to describe the situation where data from time series, literature, and stakeholder knowledge for determining parameters for simulation models are very limited (e.g. data not suitable for directly being used in SD models due to missing values or to their nonlongitudinal characteristics). We also discuss how the process of grounding the quantification of intangible variables and their relationships allows stakeholders’ involvement throughout the modelling process and increases their confidence in using the model.

The participatory SD modelling process this article is based upon involved academic experts, institutional stakeholders, and residents influenced by and influencing a major urban regeneration in Thamesmead (see Pluchinotta *et al*., 2022 and Pluchinotta *et al*., 2024 for the details on the case study). Case-study stakeholders were engaged in every step of the participatory modelling process. During the participatory process, including both a qualitative and quantitative modelling phase, the stakeholders defined the modelling focus, namely, to better understand the factors affecting the quality of public spaces (via causal loop diagram (CLD) building and analysis). They then decided to use a simulation model to identify strategies for improving the use of natural spaces in the area. Within this participatory process, the model quantification represented a challenging activity. While many quantitative SD models contain some soft variables and nonlinearities, the majority of variables are typically “tangible” or “measurable.” Our study differs in that the majority of variables representing the main issue addressed, i.e. the factors influencing people’s use of natural space, are soft variables with nonlinear relationships. In our participants’ view, the factors influencing the use of natural space involved nonlinear causal relationships with the maintenance budget as well as with “intangible variables” such as knowledge of use, use of codesign approaches, and perceived safety. The quantification of these variables and their interrelationships was often difficult, either because they proved difficult to measure or estimate because of their soft nature or because even for harder variables there was a lack of data.

To overcome such challenges, our framework suggests how to integrate diverse sources of information and adapt to context in an effective way. We sought data through review of both scientific and grey literature, targeted meetings with stakeholders, expert consultation through workshops, forms and surveys for value estimation, and influence quantification. Nevertheless, the journey faced some challenges. Here we reflect on the diversity of approaches of the stakeholders involved in the quantification of soft variables, especially when data are scarce, as well as the challenges with, for example, full stakeholder engagement, response formats, or balancing contrasting answers. We will build on our lessons learnt to suggest recommendations on the use of different quantification approaches when dealing with soft variables and data-scarce contexts.

The remainder of the article is organised as follows: after the present introduction, the section “Methods and open questions in model quantification for participatory system dynamics“ reflects on open questions in model quantification when dealing with data scarcity; the section “Quantifying a system dynamics model under different levels of data availability and stakeholder engagement“ presents our quantification process and the generic framework developed during a participatory case study for dealing with soft variables and data scarcity. Sections “Discussion: the qualitative nature of quantification“ and “Conclusions“ conclude the article with a discussion on lessons learnt and future research opportunities on the topic.

## Methods and open questions in model quantification for participatory system dynamics

This section discusses the SD quantification practices and open questions when dealing with soft and intangible variables and data scarcity during participatory processes. Several methods are available for quantifying soft variables in participatory processes. Most of them involve techniques to ask the study participants to estimate a certain parameter at a given range or giving more information on data types and availability. Specifically, modellers can directly ask the participants to estimate the variable value at a given scale. For example, Mooy *et al*. (2001) let their participants estimate variables on a “semiquantified scale” from 0 to 1. Another technique focuses not on the values of certain variables but on the relationship between these, e.g. using a “two-dimensional matrix” to asks participants to describe the effect of one variable on another variable (Mooy *et al*., 2001; Sterman, 2000). To estimate the relative influence of different factors, a swing-weighting-like technique has been used (Carnohan, 2016; Carnohan *et al*., 2016). A rigorous attempt to engage stakeholders in model quantification was made by Hosseinichimeh *et al*. (2017); namely, during Group Model Building (GMB) workshops, the facilitators identified a list of variables and units to be quantified and developed a “parameter booklet” for the stakeholders to fill out. Then the stake-holders were asked to fill the booklet with their estimation of the numeric value of variables, and the data type, availability, and sources (e.g., Andersen and Richardson, 1997; Vennix, 1996). Furthermore, qualitative techniques, not only workshops, can also support the quantification process for SD models. For instance, Luna-Reyes and Andersen (2003) described how they used interviews and activities with groups based on the Delphi method to elicit parameters from experts, and qualitative data analysis approaches such as grounded theory methods (see Glaser and Strauss, 2017) and ethnographic analysis (see Franco and Greiffenhagen, 2018) to increase the rigour of quantification. The arising attention to modelling soft and/or behavioural aspects in the last few decades provides new opportunities for embedding model quantification in GMB workshops or participatory processes; however, these attempts assumed that the engagement with stakeholders can provide enough knowledge for quantification and did not address the issue of data scarcity.

Despite the availability of methods mentioned above, there are a few open questions that demand attention when it comes to data scarcity in participatory dynamics, especially when there are abundant soft variables and/or insufficiency of relevant data that stakeholders provide through single engagement sessions. The first open question is on the difficulties of quantifying or operationalising soft variables because often they encapsulate many underlying constructs that participants bring forth. Soft variables, in comparison to “hard” variables, are often perceptions, responses, and actions that are not easily quantifiable (Checkland, 2000). Typical soft variables such as customer satisfaction of a product, trust, attitudes, etc., describe the underlying perceptions or people’s behaviour that influence the system. For example, in the World Dynamics model by Forrester (1971), the key variable “quality of life” is closely linked with crowding, pollution, hunger, illness, stress, and pressure, showing the complexity of modelling social systems. Socioeconomic or socioenvironmental systems are complex as they could include not only soft variables but also nonlinear relationships. While methods such as multipliers/effects (Sterman, 2000) can help quantify this type of nonlinear relationships, it is challenging to do when the model has a high number of soft variables and nonlinear relationships, which can be common in models developed through participatory approaches and analysing the complex interactions between e.g. the social, environmental, and technical components of a system.

The second open question relates to how a model with a majority of soft variables and nonlinear relationships, and thus a high degree of uncertainty, can lead to systems insights and what type of skills modellers need to develop for robust modelling. On one hand, the process of stakeholder engagement increases stakeholders’ systems insights and shared understanding of the problem under consideration (e.g., Pluchinotta *et al*., 2022; Rouwette *et al*., 2002; Voinov *et al*., 2018); on the other hand, simulation models, if calibrated with abundant data, allow a deeper analysis of potential policy interventions. Homer and Oliva (2001) argue that simulation models can always add value even when there is substantial uncertainty about the formulation of soft variables. The generation of useful insights also relies on modellers’ engagement and modelling skills. It has been discussed that understanding dynamic behaviours from qualitative maps requires modelling experiences “after a prolonged series of model tests of deepening sophistication and insight” (Richardson, 1996, p. 143). For instance, qualitative systems archetypes can provide insights and support in implementing systems thinking, without depending on simulation (Wolstenholme, 2003), but the development of the archetypes themselves needs years of modelling experience to connect the structure with system behaviours (e.g. Braun, 2002, described system archetypes’ behaviours).

The third open question is to what extent a participatory process is needed or useful for model quantification and what method could be used to facilitate the participatory quantification process with rigour. Confidence is critical for SD models; it addresses whether people have “confidence” in using a model to formulate policy (Lane, 2015); thus the process of eliciting quantification information is critical. Mainstream quantification practices focus on using mathematical analytical techniques to formulate and estimate the model parameters and to compare the model with historical datasets. For example, Rahmandad *et al*. (2015) describe the use of maximum likelihood estimation (a probabilistic approach to determine parameter estimation), methods of simulated moments (which use values of the moments from the simulated data as estimated value), and Markov chain Monte Carlo approaches (providing a sequence of parameters whose empirical distribution approximate posterior probability) for estimating model parameters. Indirect inference (which is a simulation-based approach to estimate parameter values) has also been used in estimating parameters when there are few data points over time (Hosseinichimeh *et al*., 2016; Hosseinichimeh *et al*., 2018). Participatory approaches for estimating parameter values can directly be influenced by stakeholders’ personal biases or the parameter value range set by the modellers due to modelling constraints. While both mathematical analytical techniques and participatory approaches have their own strengths and limitations, robust SD models often rely on the stakeholders’ knowledge of the system under consideration, and stakeholders’ numeric information of variables is equally critical as structural information of the system. As Barlas’ wrote “model validity and validation in any discipline have to have semi-formal and subjective components for several reasons often discussed in system dynamics literature” (1996, p. 183). Also, as Forrester (1994) argued, the mental database of participants who operate in the real system can generate valuable information about the system. To develop impactful, “useful,” and robust models, it therefore seems promising to integrate a participatory approach not just in the problem identification and model structure development stages but also in the quantification stage of simulation model development. In summary, despite the range of qualitative and analytical techniques available for the quantification of soft variables and nonlinear relationships, it is not sufficiently clear yet what modellers should do when facing data scarcity or when relying on analytical methods is not possible. It is also not sufficiently clear how quantification using participatory approaches can be performed with rigour.

## Quantifying a system dynamics model under different levels of data availability and stakeholder engagement

**Fig. 1:**
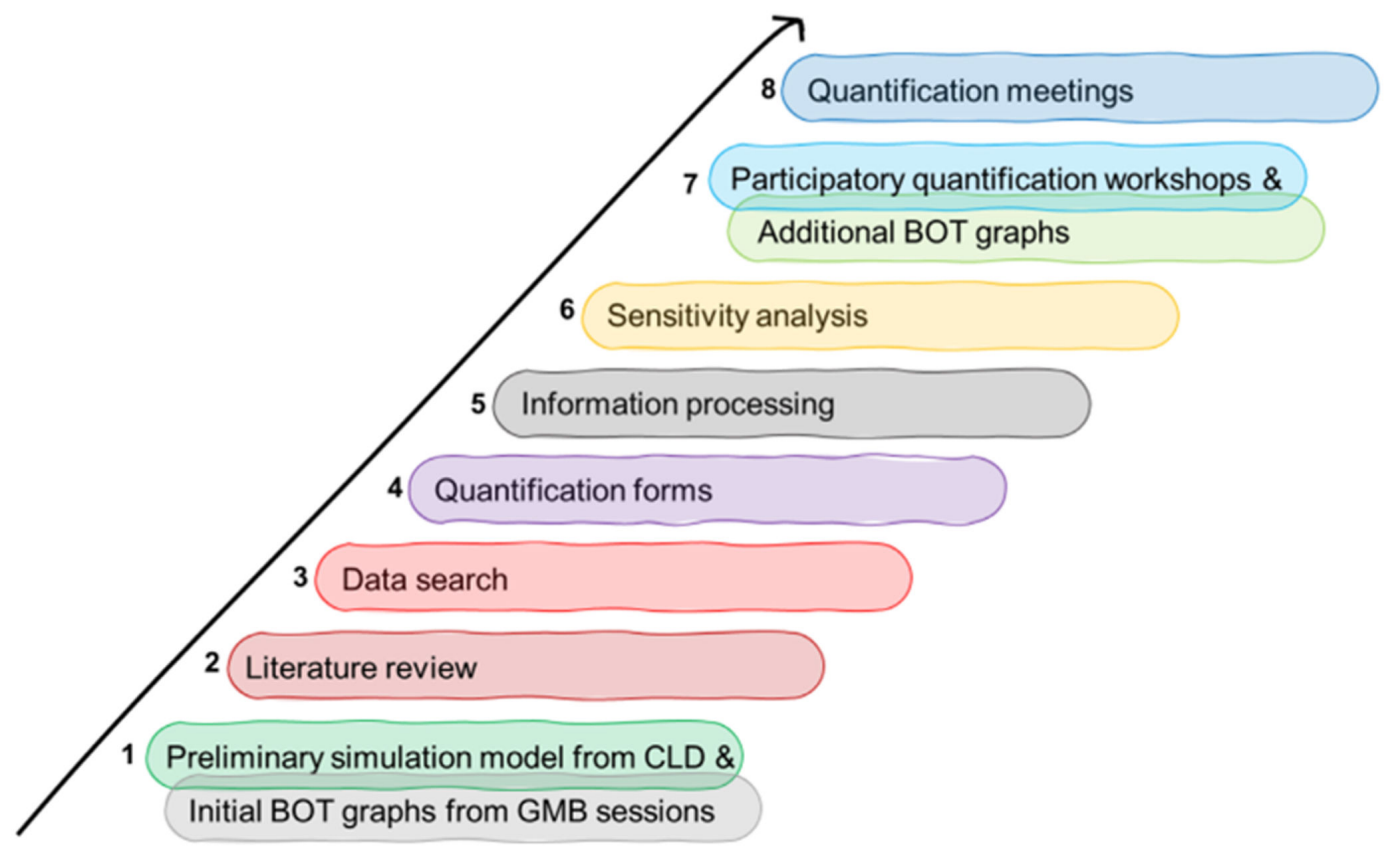
Quantification activities as carried out within the case study. BOT, behaviour over time; CLD, causal loop diagram; GMB, group model building

This section offers an overview of the quantification process carried out during a participatory SD modelling activity on the use of natural space in Thamesmead, an area undergoing urban regeneration in London, United Kingdom. Specifically, we present the developed process (8 activities listed below and summarised in Figure 1), with a focus on how we dealt with data scarcity, on the presence of a high number of soft and intangible variables, and on the quantification of their (linear or nonlinear) relationships. Furthermore, building on existing techniques, we suggest a framework (Table 3 and Figure 5) that could be used to guide quantification under different levels of data availability and stakeholder engagement.

### The quantification process of the participatory system dynamics case study in Thamesmead

The case study is part of two large-scale projects, that is, the Complex Urban Systems for Sustainability and Health (CUSSH)^i^ and the Community Water Management for a Liveable London (CAMELLIA)^ii^ projects. Through the projects, we gathered a group of diverse stakeholders, particularly from the housing, environment, and policy sector, in a transdisciplinary modelling study centred around a mutually agreed-upon focus related to sustainability and health in Thamesmead. This article will not focus on the whole modelling process; however, the qualitative modelling phase is described in detail in Pluchinotta *et al*. (2022), while the quantitative model is illustrated in Pluchinotta *et al*. (2024). Moreover, specific insights from engagement with residents are discussed in Salvia *et al*. (2022). The qualitative modelling phase had focused on the wider topic of the long-term quality of the built, blue, and green environment, and it ended with the joint agreement on the focus of the simulation model. During a collaborative process, the group of stakeholders jointly agreed that the identification of strategies to improve the *use of natural space* in Thamesmead should be the modelling focus. A simulation model was desired as it allowed the stakeholders to explore different scenarios and how different strategies may affect space use by capturing the dynamics between the influencing system elements. The model, therefore, needed to capture the main elements of the use of natural space and their interdependencies in the area under consideration, including, for instance, usability and accessibility, maintenance and space condition, residents’ perceived safety and awareness of the spaces, community participation, biodiversity, time constraints, and structural poverty. The case-study activities started in November 2019 and ended in December 2023. The quantification process was carried out from June 2020 to July 2021.

When starting to build the quantitative SD model, the team quickly realised a few challenges related to its quantification: (i) the CLD involved a large number of soft and intangible variables and nonlinear relationships between intangible and tangible variables, indicating a high level of complexity in quantification; (ii) the topic is understudied in the literature. Existing studies use conflicting definitions of urban natural space and its use, and there is limited available data on the interdependencies and relationships between the different components of this problem.

**Fig. 2:**
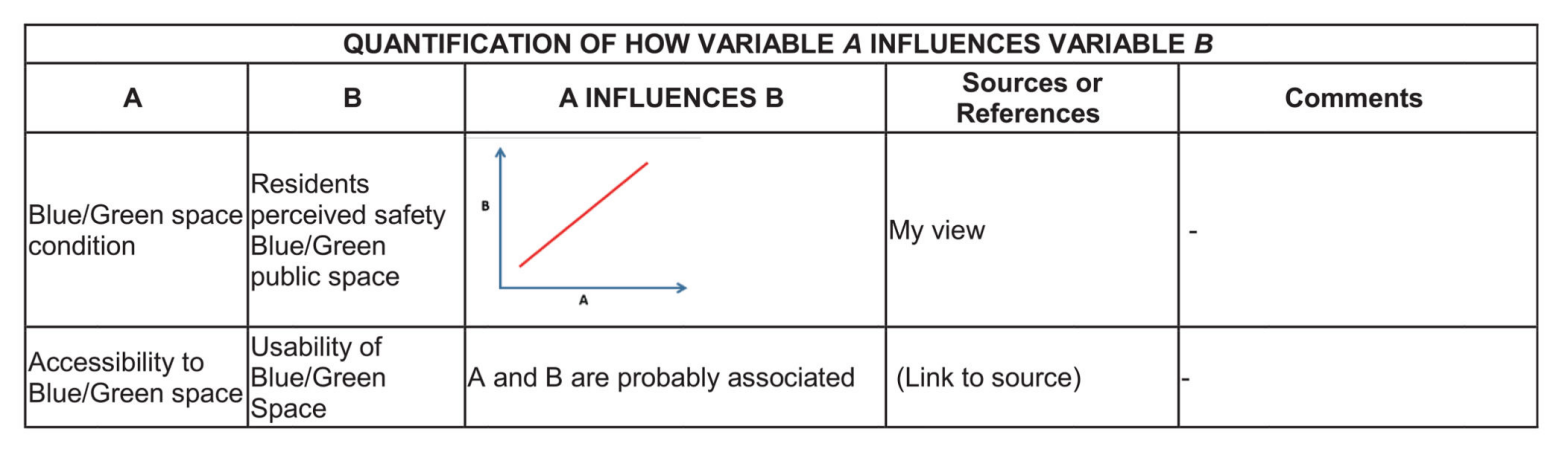
Extract from a completed quantification form for relationship quantification

**Fig. 3:**
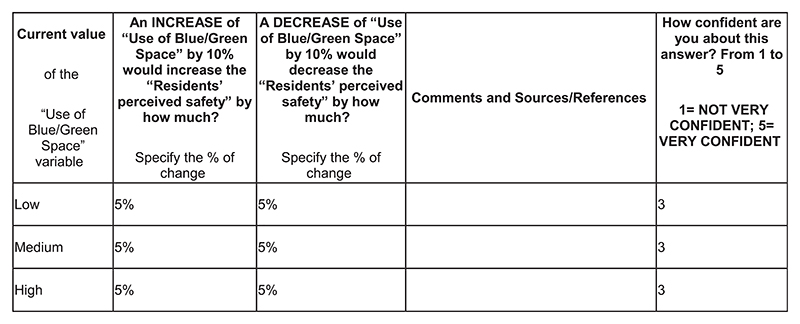
Extract from a completed quantification form for relationship estimation

To address the quantification challenges, modellers and stakeholders agreed to ground also the quantification process in a participatory approach, as this would further increase stakeholders’ knowledge on the topic and their confidence in using the simulation model (Scott *et al*., 2016). Therefore, the objective of the quantification process was to quantify model relationships, baseline values of variables, and to estimate other parameters using different sources of information, as part of a participatory modelling process. In our case study, we used the following set of activities and techniques (recapped in Figure 1):

1. Development of a preliminary simulation model following the underlying structure of the CLD built through GMB workshops. Initial behaviour over time (BOT) graphs drawn by the stakeholders are used at this stage, collected via an online adaptation of the “Graphs Over Time” script (Scriptapedia Wikibooks, n.d.).
2. Literature investigation of different model sections.
3. Dataset search to confirm data availability. This phase also includes meetings with academic experts for dataset search of key model indicators.
4. Use of online quantification forms submitted to a limited number of academic experts from domains of interest for the model under consideration. The aim of the online forms is to collect information on specific segments of the model, including variables, relationships, and weights. As example, two sections of the online quantification forms are shown in Figures 2 and 3, while the whole forms are available in sections S.1 and S.2 of the online supporting information.
5. Information processing and internal modelling sessions including domain experts when needed.
6. Sensitivity analyses to determine which variables can have the largest impact on model response. The results of sensitivity analyses allow us to further narrow down the list of items to quantify.
7. Participatory workshops for validating the model structure and collecting additional BOT graphs with the case-study stakeholders (online, 90 minutes). The agenda of the participatory quantification workshops is shown in Table 1.
8. Quantification meetings to gather specific datasets and information with relevant case-study stakeholders. A list of modelling items to discuss is prepared in advance and often shared via email beforehand. An example of the agenda for the quantification meeting is shown in Table 2.

The quantification process involved three SD modellers, four academic experts working on the case study projects (e.g., on themes of sustainability, health, codesign and urban regeneration), and 12 stakeholders, specifically eight institutional stakeholders (e.g., local authorities, environmental agency, environmental NGOs) and four members of a housing association that is considered the key decision-maker of the case study.

The researcher team built the preliminary structure of the simulation model from a jointly developed CLD, allowing us to identify parameters and specific parts of the model that needed quantification information such as variable values and relationships. During the GMB workshops, we built not only the CLD, but stakeholders also drew initial BOT graphs (activity 1). This allowed us to extract some of the baseline values from the BOT graphs when other sources of information were not available. Afterwards, we created a spreadsheet listing relationships and parameters grouped in different model sectors (~40 items). Subsequently, the SD modellers started a literature search (activity 2) using the variables and relationships under investigation as keywords and looking only at the results of the first page on one search engine, namely Google Scholar (i.e. ~10 papers per page). Then they screened literature items considering the relevance of title and abstract. They used the spreadsheet as a guide for organising the literature investigation and to report collected references and information. The objectives of this step were to identify the existence of relevant studies that looked at each specific model relationship to be quantified and, in doing so, to gather a general understanding on the specific topics included in the model. The main reason of a rapid literature search rather than a scoping or systematic review was the model size: due to the large list of items, it was not possible to carry out a detailed literature review on each model item. Relevant papers or direct paragraphs of interest were then highlighted and discussed internally. During these internal meetings, the modelling team agreed on which paper was relevant for model quantification. However, the large amount of information from the literature was difficult to prioritise. In parallel, the team carried out a search for datasets to confirm data availability and arranged discussions with experts for the identification of datasets useful for the calibration of key model indicators (activity 3). For example, we identified the UK government’s longitudinal “Monitor of Engagement with the Natural Environment” (MENE) dataset in this process, which we then used as an input to inform the quantification of the “time spent on use of space” sector and from which colleagues from the CUSSH project helped generate the final BOT for calibrating the model. The MENE survey has been conducted from 2009 onwards by Natural England in partnership with the Department for Environment, Food and Rural Affairs (Defra) of the UK government (Natural England, 2019). The national-level survey asks respondents about the number of visits to natural areas over the previous 7 days and the total visit duration in minutes. We extracted data on all respondents residing in the case study area, as identified by the postal codes.

Considering the large number of nonlinear relationships and soft variables, and the scarcity of literature and evidence on the case study topic, ~25 items still needed to be quantified. Therefore, the team decided to prepare tailored quantification forms paired with a detailed explanation email for 11 academic experts (activity 4). The academics were involved in the projects, and they were selected by their field of expertise and relevance to the variables that need to be quantified. It is worth clarifying that case-study stakeholders were involved in the quantification process, as much as in the modelling one; however, since they often had limited knowledge on key components of the model, we included topic experts from academia (e.g., the natural spaces condition was quantified via an interactive spatial map pointed out by an expert, or the effect of safety-design aspects on the residents’ perceived safety was estimated by experts of the sector). The academic experts were involved with the specific task of providing knowledge on specific components of the model (e.g., using online forms; step 4 of the quantification process). We organised six additional meetings with some of the experts and also sent follow-up emails. We sent the tailored quantification forms at the same time to all the experts, and they consisted of an introduction with instructions on how to fill in the form, a short model description including the CLD behind the model, and four sections asking: (i) for units, comments, and references for the model stocks (depending on expertise), for which we provided definitions for context, (ii) to provide variables’ possible baseline and goal values within the case study or in similar contexts with references (depending on expertise), (iii) to provide references and comments to help quantify specific model relationships (namely, how variable A influences variable B), (iv) for their own estimate of the strength and nature of relationships in case any other references were not available (estimates how variable A influences variable B under different conditions). We estimated it would take 60–90 minutes to complete the form, but explained to the participants that more detailed answers could take more time. See Figures 2 and 3 and the online supporting information (S.1) for examples of the quantification forms submitted to the experts. Mainly due to the lack of time for establishing new academic collaborations, resources for completing the form, understanding of the model and modelling process, only five forms were returned.

A second form was sent to relevant academic experts to quantify weighted equations. Generally, these equations represented the grouped effect of a set of variables on a certain variable. Then, the modellers mathematically aggregated individual inputs and calculated the weights from the ranks using established methods from the academic literature (e.g., Chalabi *et al*., 2017, and Kenyon, 2007).

Subsequently, the SD modellers processed the large amount of information received thanks to the forms and related interaction with the academics (activity 5). Despite the tailored forms and the instructions provided, the information provided was not in an easily and directly quantifiable format; the colleagues pointed us to key papers they were aware of rather than providing values. While a summary of all the suggestions was under development, the modellers realised that it was difficult to prioritise information and adapt it to the model. They then held internal discussions for modelling decisions. Subsequently, the SD modellers performed sensitivity analysis and discussed results among the team (activity 6) testing the exogenous variables and “effects” variables. Scales of the value and strength of impact were established. The output of the sensitivity analysis was a narrowed list of variables that have large impact on the model results.

Lastly, the modelling team organised two online “quantification and validation” workshops with institutional stakeholders and members of the housing association to collect the last missing data (between four and eight participants) (activity 7). The workshop objectives were (i) to present and further validate the structure of the SD model and (ii) to help quantifying the SD model using more BOT graphs for soft and intangible variables (e.g., perceived safety in urban natural space, residents’ awareness of opportunities in natural space, community participation). The scope of the BOT activity was to describe the change over time of a selection of variables related to the case study topic and to collect more knowledge and information of the selected variables. Variable definitions and units were provided, and the workshop participants were able to draw behaviour over time and comment on it using an online open-source digital interactive white-board (Jamboard developed by Google). See Table 1 for the workshop agenda. To conclude the quantification process, meetings were then organised with the case-study stakeholders to gather further datasets and missing information relevant for specific parts of the model (see Table 2 for an example of a meeting agenda).

While we recognise the benefits of a participatory approach with the model focus shaped by the stakeholders, the overall quantification process was resource demanding. It could have been speeded up by: (i) an even higher degree of shared responsibility for quantification among the wide research group (beyond the SD modellers team); (ii) access to subject matter experts on each and every variable with time allocated to support modelling; (ii) if we could have encouraged experts to provide estimates more comfortably when they could not give fully accurate answers.

As a practical example, the following section presents a segment of the case-study model showing which methods we used to quantify each shown variable, underlining the different sources of knowledge and information and the approaches used.^iii^

### Quantification example: different sources of information and knowledge

**Fig. 4:**
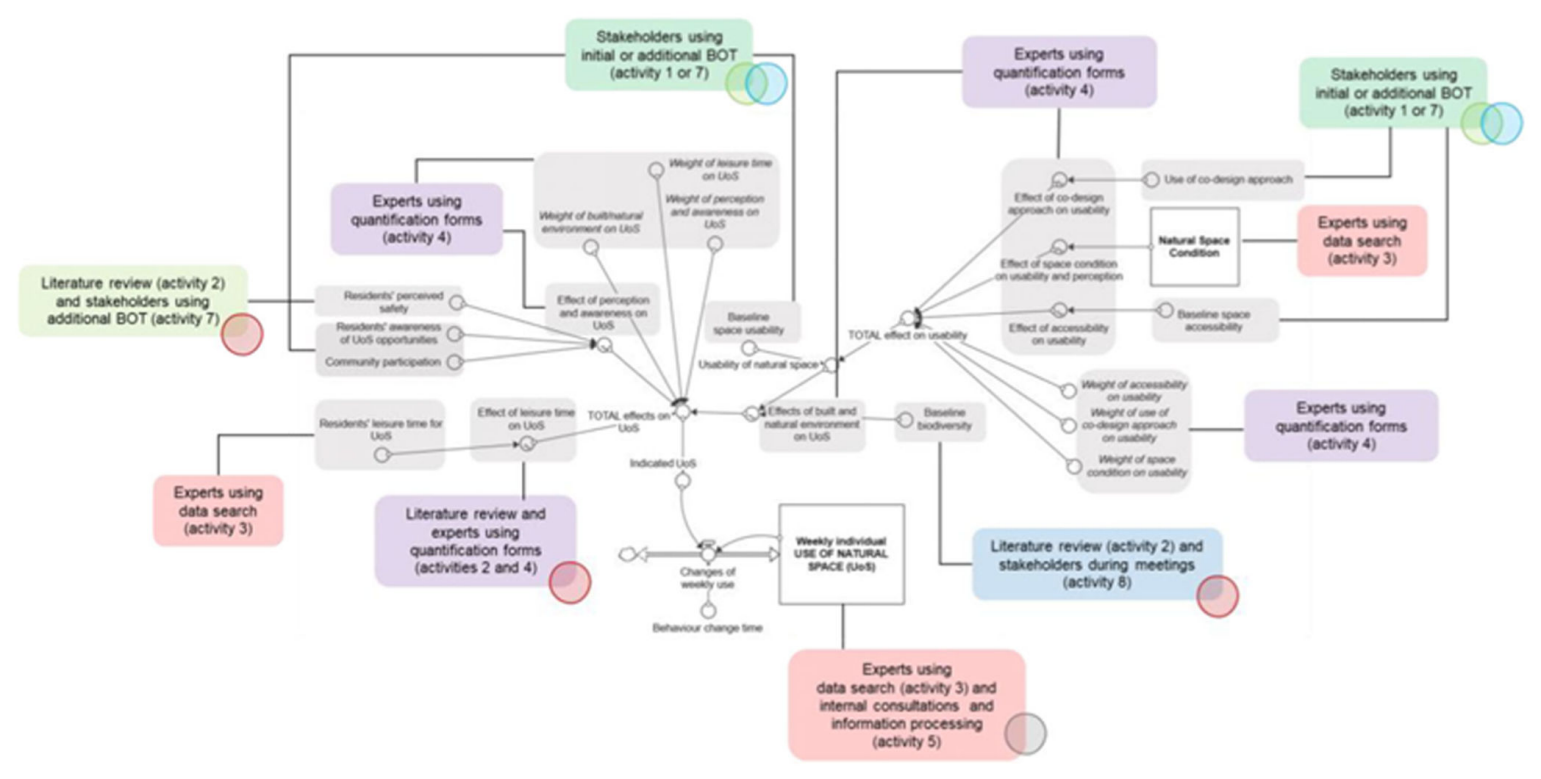
Segment of the model with additional information on information sources for quantification

Figure 4 presents a segment of the model and respective quantification sources, providing an example of how we used different sources of information and knowledge to quantify the model under consideration. Use of natural space (UoS) is the key stock in the model (“Weekly individual use of natural space (UoS)” with units “visits/week/person”), which is shown at the centre bottom of Figure 4, indicating the amount of the weekly use of space in the case-study area. It is an exponential smooth of the “Indicated UoS,” influenced by the “Behaviour change time” (defined as the time needed to change the UoS behaviours). Specifically, the “Indicated UoS” depends on three elements: (i) perception and awareness-related components which include the variables “Residents’ perceived safety,” local “Community participation,” and “Residents’ awareness of UoS opportunities,” i.e. of opportunities for how to use the space; (ii) built/natural environment components influencing the use which include the variables

“Usability of natural space” and “Biodiversity”; and (iii) “Residents’ leisure time for UoS” (i.e. how much time people have for using the natural spaces). We elicited these model elements in different ways (see Figure 3); for instance, we elicited the baseline value and estimated changes after the regeneration of the area from case-study stakeholders through our participatory workshops (activities 1 and 7 of the quantification process). We modelled the specific “effects” of how the use is influenced as graphical functions and directly consulted experts via the quantification forms (activity 4). The results of the literature review (activity 2) were often integrated to the discussion. Experts also estimated the weights of each weighted variable through individual rankings of the importance of variables. The variable “Residents’ leisure time for UoS” is modelled as the average duration of monthly outdoor visits to spaces (in minutes). For the purpose and boundaries of the model, leisure time is interpreted as the time available to spend in public natural space rather than in general. We imported data from the Monitor of Engagement with the Natural Environment (MENE) survey (Natural England, 2019) (activity 3).

In summary, the UoS model captures a number of variables including residents’ perception, awareness, time, and the complex pathways of how the built environment and natural environment impact people’s use of natural space. The integration of information, such as baseline value estimation, weights and graphical functions from stakeholders, experts, and empirical datasets shows the complexity of the quantification process when dealing with data scarcity and an abundance of soft variables.

### The quantification framework

**Fig. 5:**
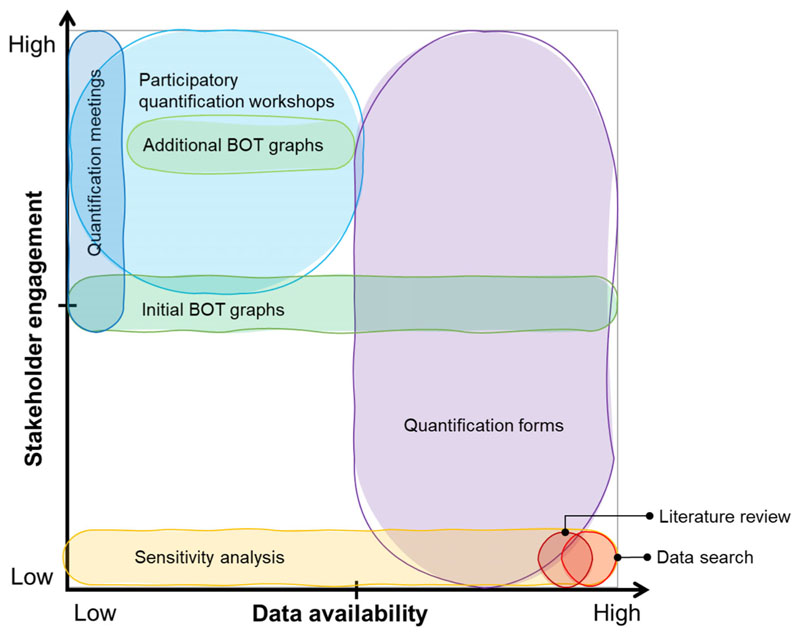
Generic quantification framework relating potential quantification procedures to levels of data availability and stakeholder engagement BOT, behaviour over time

After reflecting on the process carried out and how we tried to deal with the challenges of quantifying qualitative information under data scarcity, we suggest the following practical quantification framework (described in Table 3 graphically presented in Figure 5). Building up from existing methods and techniques and adapting them under an operational perspective, Table 3 includes quantification activities that modellers can select when quantifying an SD model under different levels of data availability and stakeholder engagement. Vice versa, in a participatory process, the framework underlines what the possible quantification activities are. For instance, from Table 3, in case of high data availability and low engagement, the framework suggests the use of literature review and dataset search. In case of a medium level of stakeholder commitment, the online qualification forms could help in gathering useful and well-identified information in a structured way.

Lastly, quantification meetings and workshops are effective activities in case of low data availability and a higher level of stakeholder involvement. Since it is not always easy to identify the right method to use when quantifying a model, we advise structuring the quantification process according to the degree of stakeholder engagement and data availability, starting with methods appropriate for high data availability and moving down as required.

As further represented in Figure 5, the generic framework (Table 3) relates potential quantification procedures to levels of data availability and stakeholder engagement, providing guidance on a range of existing methods, that could be used in different contexts in which modellers might find themselves. Our framework suggests how to integrate diverse sources of information and adapt to context in an effective way, providing the reader with examples of activities. In other words, from Figure 5, when data availability is high (i.e. when it is possible to find the needed data for qualification from literature or databases), then the modeller may be able to quantify the variables without stakeholder engagement. When data availability is medium to high (i.e. when the modeller cannot obtain qualification data from literature review or database search), it could be particularly useful to consider online qualification forms. This would involve varying levels of engagement with stakeholders, who are typically the topic experts. Activities such as quantification or workshops or drawing additional BOT graphs are particularly useful for lower levels of data availability. Drawing initial BOT graphs and performing sensitivity analysis is useful at any level of data availability. Namely, as in the case-study context, the presence of soft variables and data scarcity do not allow the use of only one quantification activity. It may also be necessary to use different quantification strategies for different variables or elements of a model.

## Discussion: the qualitative nature of quantification

### Modelling the issue rather than modelling where data are available: difficulties inoperationalising soft variables

Forrester (1992) emphasised that, if we reduce our modelling efforts to the area where we have quantitative data available, we capture only a small fraction of the real world and of the information that influences people’s decision-making, routines, and actions. Indeed, the often-explorative nature of SD models is one of the key advantages of SD, even though it also represents a challenge for quantifying the qualitative aspects, especially when it is not possible to apply conventional analytical methods. SD has thus always relied on a breadth of information for building models. By modelling the use of natural space in Thamesmead, we entered an area where not only little to no quantitative information was available but where even mental models of certain past trends or of effect strengths were vague. For instance, our stakeholders preferred not to provide their own perceptions during workshops, when asked to compensate for the lack of data from literature or datasets; they would have preferred to be told numbers, yet these did not exist. Emphasising that this information is not readily available, that an estimate is better than no information, and that we would triangulate their responses with other forms of data helped address such reluctance. Thus, the model’s structure is grounded in the mental models of our diverse case-study stakeholders, workshop participants, and interviewed residents, and the model’s parameters are grounded in a true mix of data from literature, expert opinion elicited via various means as well as modelling logic. This makes it a quantitative model of rather conceptual nature that shares similarities with models built for theory development, such as the one by Sastry (1997) and Zimmermann (2011) or discussed by de Gooyert and Größler (2018) and de Gooyert (2019). It also sits between illustrative models with limited details but plausible scaling, which are very typical for SD modelling and metaphorical or exploratory models with minimal details and grounding in quantitative data, which provide insight into the “inner workings” and properties of the system under consideration (Homer, 1996, 2014; Morecroft, 2015; Richardson, 2024). As underlined by Morecroft (2012), a model, though stylised, needs to be both plausible and sufficiently understandable to stimulate comparisons with the real world, and, therefore, key assumptions of the model should be clear and recognisable.

### How a quantitative model of conceptual nature can be used to lead to systems insights

Being a quantitative model of rather conceptual nature has implications for the model’s use, mainly due to substantial modelling assumptions and the related high level of uncertainty. Typically, SD models are built not for predictive purposes but for enhancing people’s understanding of the system, to identify leverage points, to support strategic thinking on the problem, or to identify areas that possible solutions should consider. With quantitative models of conceptual nature, this is even more pronounced. The model, its interface, and the simulation outputs can be used as a boundary object to trigger discussion among a group of stakeholders or externals (e.g. Black, 2013; Luna-Reyes *et al*., 2019; Pluchinotta *et al*., 2018), to make them reflect on their assumptions (e.g. Eker *et al*., 2018), to create interdisciplinary learning, to increase and guide communication across a wider range of stakeholders, and to support a collaborative setting. In our concrete case, some of the simulation runs revealed unexpected leverage and synergies between maintenance practices and the use of codesign approaches with the local residents, which — while not being reliable in their absolute effects — increased the stakeholders’ willingness to investigate whether and how they want to invest more resources into these areas, overcoming differences of strategic objectives of different stakeholder organisations. In detail, from using the model to simulate scenarios and test strategies, stakeholders realised that maximising the design aspect of the built environment (accessibility and safety) alone does not have major impact on improving the use of natural space in the area (desired goal). Instead, strategies focusing on community interventions and codesign combined with the extra needed maintenance have larger influence on the use of the space. Therefore, stakeholders used the model to investigate reasoning, helping the collaboration and learning of all the parties involved.

A model built on scarce data embodies a great deal of uncertainty, and therefore the output of such a model will necessarily need to be interpreted carefully. It cannot be used to answer all the questions that stakeholders may have in an area where research and data are scarce. SD models are effective in supporting decision-making at a strategic, system-wide level through collaborative discussion, to ultimately enable the exploration of long-term consequences of alternative strategies, particularly those that are difficult to include in purely quantitative models (e.g. Pluchinotta *et al*., 2021). However, we could clarify this with the stakeholders and manage their expectations, and their understanding of the system improved thanks to the modelling process and results, as well as interactions with others involved. A quantitative model of a conceptual nature can nevertheless direct other research to the most urgent or fruitful areas, for example, a focus on levers or newly discovered unintended consequences, In our specific case, these are the effect of the use of natural space to the heath of the local population (and vice versa), the role of deprivation as barrier to the use of space and also as key influence to the perceived safety of the area, the effect of the use of codesign approaches as a booster of the use of space from the local residents, which all need further research beyond our and existing studies on the question of how to enhance people’s use of natural space.

Our case study is an example for how qualitative the nature of the quantification process can be. It goes beyond behavioural validation and includes large elements of judgement by experts, stakeholders, and researchers. This judgement concerns which method(s) to use for quantification, whom to involve for expert/stakeholder judgements, and how to deal with conflicting responses by the consulted individuals. Critical systems approaches emphasise that these judgements involve elements of power because they affect who is able to provide their perspective (Reynolds and Holwell, 2020).

### How to facilitate the participatory quantification process with rigour: lessons learnt

All models represent a simplification of reality, and their quantification is influenced by, among other factors, the modelling objective, data availability, and limitations imposed by time and logistical constraints (e.g. Pluchinotta *et al*., 2021). As underlined in the previous sections, some quantification practices have been shared, but it is not always easy to identify the right method to use when quantifying a model in a participatory way and under numeric data scarcity. To overcome the difficulties incurred, the team used a range of methods more or less known in the SD literature and described their composition.

The preliminary CLD and respective BOT graphs were crucial in guiding the quantification efforts. The spreadsheet listing everything that needed to be quantified gave us the necessary overview for managing the entire quantification process and to start the literature search. The latter together with datasets, where available, helped ground the model and provided a means to triangulate with data elicited in a participatory way. Quantification forms particularly helped in quantifying model elements for which data were scarce. However, spending more time in presenting the model and clarifying our data needs would have better steered the respondents to what we were seeking. Iterative steps of information processing as well as sensitivity analyses were needed to manage the multitude of information, of possible directions, and to guide the next steps. Participatory quantification workshops with additional BOT graphs turned out crucial particularly in areas where literature and datasets were scarce as well as in supporting the model’s relevance for stakeholders. Limitations and difficulties to overcome in each activity of the quantification process are outlined in the section “Quantifying a system dynamics model under different levels of data availability and stakeholder engagement“ with a description of the modelling issues that one might have to deal with when there is the need of quantifying a model with a majority of soft variables and nonlinear relationships and consequentially with a high level of uncertainty.

We recommend structuring the quantification process according to the level of stakeholder engagement and data availability, starting with methods appropriate for high data availability and moving down as required. The framework provides guidance on possible methods and activities that could be used to collect knowledge and data.

## Conclusions

This article tried to shed light into a common issue encountered by SD modellers, namely, how to deal with data scarcity and soft variables when quantifying a model. While the SD community has long emphasised the value to focus modelling on the most important issues rather than on where the data are, this article takes this very idea further to areas where there is not just some data missing or uncertain and where there are soft variables, but where gaps, uncertainties, and soft variables exist in abundance. Resulting models share many aspects with models of theory, metaphorical or conceptual models, requiring even further carefulness in the interpretation of quantitative results and their communication to stakeholders. One may argue that it may be better to stick to qualitative, i.e. nonsimulation models if faced with such gaps and uncertainties, yet we would argue against that, and we showed how insights on the relation of structure and behaviour can still emerge and how policy insights may still be possible when underpinned with multiple scenario and sensitivity tests. Our framework mapped different quantification methods against data availability, and the process we used in quantification could be copied or modified by others (Figure 5). The framework helps the modellers to come up with an appropriate strategy to quantify an SD model. The nonparticipatory activities might be applicable to any quantification process, since participatory modelling and participatory quantification are different (e.g. an initially nonparticipatory modelling project could include participatory quantification). Furthermore, the framework shows the breadth of data we can use to quantify a model and strategies we can employ when numerical data are not available. This is very valuable, especially for beginner SD modellers. We hope that our contribution could lead to future interest and discussions on this key phase of the modelling process, resulting in an even larger range of quantification methods.

## Supplementary Material

Supporting information

## Figures and Tables

**Table 1:** Participatory quantification workshop agenda

Participatory quantification workshop (90 min)	
*Aims:* to present and validate the structure of the SD simulation model, to help quantify specific sections of the model	
*Location:* online (Microsoft Teams)	
Time	Activity
5 minutes	Welcome and workshop objectives
15 minutes	Presentation of a simplified version of the SD model using a CLD when needed, including model assumptions and calibration process
25 minutes	Structured discussion for validating the model structure
5 minutes	Introduction of the drawing BOT graphs activity
25 minutes	BOT activity for a selection of variables. Stakeholders are divided in groups
10 minutes	Presentation of the BOT graphs created by the groups
5 minutes	Next steps and closing

**Table 2:** Example of quantification meeting agenda

Quantification meetings (~60 minutes)	
*Aim:* to gather specific datasets and information with relevant case-study stakeholders *Location:* online (Microsoft Teams) *Note:* A list of modelling items to discuss is prepared in advance and often shared via email beforehand.	
Time	Activity
5 minutes	Welcome and meeting objectives
10 minutes	Recap of the modelling activity using a simplified version of the SD model
40 min	Discussion for collecting specific information and requesting access to datasets relevant for the model quantification.
5 minutes	Next steps and closing

**Table 3:** The generic quantification framework showing different methods and activities depending on the degree of data availability and level of stakeholder engagement

Quantification activities	Level of Data availability	Level of Stakeholder engagement	Type of engagement	Purpose
Initial BOT graphs building	Any type of data availability	Medium. It involves an active GMB workshop participation	Engagement with local stakeholders and experts	Identify structural elements that need quantification
Literature review	High	None	-	Determine relationships among structural elements that will ultimately be represented by formal equations in the simulation model
Dataset search	High	None	-	Identify existing data to support formulation of specific equations, reference modes and parameter values
Online quantification forms	Medium to high	From low to high. It depends on the time spent for filling in the online form	Engagement with academic experts in specific topics	Elicit data from experts to address data gaps (on reference modes, parameter values and equations)
Sensitivity analysis	Any type of data availability	None	-	Evaluate importance of any missing data and identify high priority data sourcing needs
Quantification and validation via participatory workshops including additional BOT graphs	Low to medium	Medium to high. It involves an active workshop participation	Engagement with local stakeholders	Seek to fill high-priority data needs using stakeholders’ knowledge and mental models, gain stakeholder support
Quantification meetings	Low	Medium to high. It implies answering to specific questions and providing data and information during and after the meeting	Engagement with local stakeholders with a specific type of knowledge useful for a specific model section	Seek to fill high-priority data needs using stakeholder’s knowledge and mental models

Abbreviations: BOT, behaviour over time; GMB, group model building.

## Footnotes

i https://projectcussh.org/ and https://www.ucl.ac.uk/complex-urban-systems/cussh.

ii https://www.camelliawater.org/.

iii Since this article focusses on model quantification, we do not consider the inclusion of the whole model to be of relevance. However, the model is fully described in Pluchinotta *et al*. (2024), and it is available at https://exchange.iseesystems.com/public/ucl/thamesmead-use-of-natural-space-model/index.html#page1. The authors worked on a second version of this model after submitting the present article. The new version explores the effects on health of the use of urban natural space: https://exchange.iseesystems.com/public/ucl/thamesmead-use-of-natural-space–health/index.html#page1.
