# Supplementary material for: Dealing with soft variables and data scarcity: lessons learnt from quantification in a participatory system dynamics modelling process
Source: Syst Dyn Rev. Author manuscript; Available in PMC 2024 Oct 21. (PMC7616723; doi:10.1002/sdr.1770)
Supplement: Supporting information [file EMS199301-supplement-Supporting_information.docx]

**Dealing with soft variables and data scarcity: lessons learnt from quantification in a participatory system dynamics modelling process**

Irene Pluchinotta*, Ke Zhou, Nici Zimmermann

Contact: [i.pluchinotta@ucl.ac.uk](mailto:i.pluchinotta@ucl.ac.uk)

**Supplementary Materials**

This section includes additional details related to the quantification process described in Section 3 of the main manuscript.

**S.1. Example of the quantification form for variables and relationships submitted to the case study experts**

**Model overview:**

During the last participatory workshop, Thamesmead (TM) stakeholders jointly agreed on the development of a System Dynamics (SD) model describing the use of urban natural (Blue/Green, BG) spaces. The simulation model is based on an aggregate qualitative Causal Loop Diagram describing the main aspects of the use of BG spaces. According to the stakeholders’ understanding, the use of the BG spaces is linked to: (i) Usability of BG spaces (i.e., features of the physical space, such as no. of facilities, and accessibility); (ii) Residents’ awareness of opportunities, (iii) Community participation and stewardship, (iv) biodiversity and more generally natural space performance, (v) Time constraints and structural poverty, (vi) Maintenance and space decay, and (vii) Residents’ perceived safety.

**Purpose of this exercise:**

We are now quantifying the “influences”, i.e., the linear/nonlinear relationships in the model, consulting the project team. Thus, the main goal is to quantify how one variable of the model influences the changes in another variable of the model. We are asking you to provide us with references to studies, whenever you can. For those influences that have not been fully studied, we would like to obtain your best judgment concerning estimated effects.  We thank you in advance for taking the time to contribute to an important aspect of this study.

**What we need:**

**Question 1** - **Stocks quantification:** **Could you please fill-in Table 1?** We are asking your opinion on the most appropriate unit for each stock, but also the current value and desirable goal in TM, if known. If you have any knowledge of papers we should read, please always indicate that in the related cell.

**Question 2** - **Variable quantification:** **Could you please fill-in Table 2?** We are asking your opinion on the most appropriate unit for the variables in your area of expertise. If you have any knowledge of papers we should read, please always indicate that in the related cell.

**Question 3 – Relationship quantification**: **Could you please fill-in Table 3?** If you have any knowledge of papers we should read or literature we should review, please always indicate that in the related cell.

**Question 4 – Relationship estimation - Estimates how variable *A* influences variable *B* under different conditions: Could you please fill-in Table 4?** There is one section of the table for each relationship under consideration.

*Supplementary Materials Table 1. Stocks quantification*

| **Stocks** | **Description** | **Units** | **Current value**in Thamesmead  (or in a similar neighbourhood, please specify) | **Goal** in Thamesmead  (or in a similar neighbourhood, please specify) | **Sources or References** | **Comments** |
| --- | --- | --- | --- | --- | --- | --- |
| Blue and Green (BG) Spaces condition | Current level of quality of the BG spaces. It is influenced by the maintenance practices | % | See link | Not sure | (Link to source) | I found this green space rating website very useful  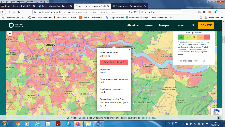 |
| Usability of BG spaces | Level of the usability of BG spaces, which is influenced by connectivity and accessibility, design approaches and space condition | % | See link | 90-100% | (Link to source) | - |
| … | … | … | … | … | … | … |
| Provided by the modelling team | | Filled in by the expert | | | | |

Supplementary Materials Table 2. Variable description and units

| **Variables** | **Description** | **Units** | **Comments** |
| --- | --- | --- | --- |
| Accessibility of BG spaces | The level of accessibility of BG spaces. It is influenced by availability of public/private transports, physical obstacles and challenging typography of paths, and level of access standards | % | “only 18 per cent of London’s green spaces accessible to the public”  (Link to source) |
| Community participation | The level of residents participating and stewarding to the regeneration of the local BG areas. It is based on the percentage of residents that are aware of local regeneration after a delay of time, it is influenced by the time residents have available to participate engagement events, level of environmental education, and the actual use of space | % of eligible residents | I am assuming some residents are ineligible (e.g. infants or the infirm). I am also assuming that there is no ceiling, i.e. there is capacity for 100% of eligible residents to participate in stewarding. However perhaps this is not realistic. |
| … | … | … | … |
| Provided by the modelling team | | Filled in by the expert | |

Supplementary Materials Table 3. Relationship quantification

| **QUANTIFICATION OF HOW *A* INFLUENCES *B*** | | | | |
| --- | --- | --- | --- | --- |
| **A** | **B** | **A INFLUENCES B** | **Sources or References** | **Comments** |
| BG spaces condition | Residents perceived safety of green and public spaces | 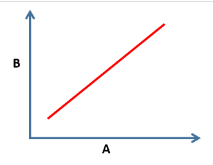 | My view | - |
| Accessibility to BG spaces | Usability of BG Spaces | A and B are probably associated | (Link to source) | - |
| … | … | … | … | … |
| Provided by the modelling team | | Filled in by the expert | | |

Supplementary Materials Table 4. Relationship estimation

| **Current value**  of the  “Safety-design aspects” variable | **An INCREASE of “Safety-design aspects” by 10% would increase the “Residents’ perceived safety” by how much?**  Specify the % of change | **A DECREASE of “Safety-design aspects” by 10% would decrease the “Residents’ perceived safety” by how much?**  Specify the % of change | **Comments and Sources/References** | **How confident are you about this answer? From 1 to 5**  **1= NOT VERY CONFIDENT; 5= VERY CONFIDENT** |
| --- | --- | --- | --- | --- |
| Low | 25% | 25% |  | 3 |
| Medium | 25% | 25% |  | 3 |
| High | 25% | 25% |  | 3 |
| **Current value**  of the  “Use of BG Spaces” variable | **An INCREASE of “Use of BG Spaces” by 10% would increase the “Residents’ perceived safety” by how much?**  Specify the % of change | **A DECREASE of “Use of BG Spaces” by 10% would decrease the “Residents’ perceived safety” by how much?**  Specify the % of change | **Comments and Sources/References** | **How confident are you about this answer? From 1 to 5**  **1= NOT VERY CONFIDENT; 5= VERY CONFIDENT** |
| Low | 5% | 5% |  | 3 |
| Medium | 5% | 5% |  | 3 |
| High | 5% | 5% |  | 3 |
| … | … | … | … | … |
| Provided by the modelling team | Filled in by the expert | | | |

**S.2. Example of the quantification form for weights elicitation submitted to the case study experts (weighted equations to represent grouped effects)**

Please order the following variables from the most influential (1) to the least one (3)

| **Variables influencing Usability** | |
| --- | --- |
| **Variables** | **Individual Rank** |
| Use of co-design approaches |  |
| Accessibility |  |
| Space condition |  |

Please order the following variables from the most influential (1) to the least one (3)

| **Variables influencing Use of space** | |
| --- | --- |
| **Variables** | **Individual Rank** |
| Residents’ perception of safety and awareness of opportunities on the use of spaces |  |
| Resident’s leisure time |  |
| Built/natural environment components (i.e. Biodiversity, Usability) |  |

Please order the following variables from the most influential (1) to the least one (4)

| **Variables influencing Biodiversity** | |
| --- | --- |
| **Variables** | **Individual Rank** |
| Active travel |  |
| Use of space |  |
| Community participation |  |
| Maintenance |  |
